# Mechanistic Insights Into GDFMD-Mediated Inhibition of Liver Fibrosis via miRNA-29b-3p Upregulation in Wilson's Disease

**DOI:** 10.1155/mi/2776808

**Published:** 2025-04-26

**Authors:** Peng Huang, Yuzhe Huang, Ting Dong, Han Wang, Meixia Wang, Xiang Li, Wei Dong, Yulong Yang, Wei He, Wenming Yang

**Affiliations:** ^1^Department of Neurology, The First Affiliated Hospital of Anhui University of Chinese Medicine, Hefei, Anhui, China; ^2^Anhui University of Chinese Medicine Key Laboratory of Xin'an Medicine of the Ministry of Education, Hefei, Anhui, China; ^3^School of Pharmacy, Anhui University of Chinese Medicine, Hefei, Anhui, China

**Keywords:** autophagy, hepatic fibrosis, hepatic stellate cells, myofibroblasts, Wilson's disease

## Abstract

**Background:** Wilson's disease (WD) is an abnormal copper metabolism disease. GanDouFuMu decoction (GDFMD) is a traditional Chinese medicine, whicn has shown good therapeutic effects in clinical treatment of WD liver fibrosis;but its regulatory mechanism is still unclear.

**Methods:** The serum of WD patients before and after GDFMD treatment were collected, the four items of liver fibrosis were detected by ELISA. The hepatic stellate cell (HSC) activities were assesed via CCK8 assay. The mRNA levels were evaluated by qPCR. The protein levels were checked by western blot. The autophygosomes were observed by transmission electron microscope (TEM). The transdifferentiation ability of HSCs into myofibroblasts was evaluated with anti-α-SMA antibody by immunofluorescence (IF). In copper-laden rats with WD, the autophagy levels, and fibrosis level were observed by IF.

**Results:** The four items of liver fibrosis levels were decreased. GDFMD could increase the HSCs cell activity. GDFMD could increase miRNA-29b-3p levels, which was decreased by TGF-β1. miRNA-29b-3p inhibitors could reversed the suppression response of GDFMD on the the protein expression of ULK1, beclin1, LC3, α-SMA, and Col1. GDFMD blocked the transdifferentiation of HSCs into myofibroblasts, inhibited liver fibrosis.

**Conclusion:** GDFMD blocked the transdifferentiation of HSCs into myofibroblasts by upregulating miRNA-29b-3p, and then inhibited liver fibrosis in WD.

## 1. Introduction

Wilson's disease (WD), also called hepatolenticular degeneration, is a potentially treatable, inherited disorder of copper metabolism, which is characterized by the pathological accumulation of copper [1]. WD is caused by mutations in ATP7B gene, which encodes a transmembrane copper-transporting ATPase. ATP7B gene mutation leads to impaired synthesis of ceruloplasmin and dysfunction of copper excretion in bile, which leading to impaired copper homeostasis and copper overload in the liver, brain, and other organs [2]. The typically reported prevalence of WD of 1:30000. The highest being 885 per million from within the mountainous region of Rucar in Romania. In Sardinia, where the prevalence of WD has been calculated at 370 per million births, six mutations account for around 85% of WD chromosomes identified [3]. Using this most conservative approach, the calculated frequency of individuals predicted to carry two mutant pathogenic ATP7B alleles is 1:7026 [4]. Liver is the central organ of copper metabolism. WD liver fibrosis is a pathological process of reversible deposition of extracellular matrix (ECM) after chronic liver injury caused by copper ions. The main cause of death in patients with WD is cirrhosis and its complications [5]. WD liver fibrosis has become an urgent clinical problem to be solved.

Hepatic stellate cells (HSCs) are resident nonparenchymal liver pericytes whose plasticity enables them to regulate a remarkable range of physiologic and pathologic responses. HSCs could regulate carbohydrate, mitochondrial, lipid, and retinoid homeostasis [6]. In chronic liver injury, HSCs drive hepatic fibrosis process. HSCs are activated from a quiescent state into proliferative. Activated HSCs are transdifferentiate into motile myofibroblasts, given their potential to induce connective tissue formation and ECM protein accumulation [7, 8]. Retardation of proliferation and clearance of activated HSCs from the injured liver is an appropriate therapeutic strategy for the resolution and treatment of hepatic fibrosis [9]. In the treatment of expelling copper, the metal chelating agent based on penicillamine is often forced to stop for its severe toxic and side effects. According to Xin'an theory of traditional Chinese medicine, GanDouFuMu decoction (GDFMD) was created. The clinical monitor found GDFMD can effectively inhibit liver fibrosis, improve the symptoms of patients and related biochemical indicators, so as to improve the quality of life of patients [10].

As endogenous, regulatory, single-stranded, noncoding RNAs, microRNAs (miRNAs) negatively modulate gene expression by either promoting the degradation of mRNA or downregulating the protein production by translational repression. miRNA-29 is recently discovered as a class of miRNAs which is related to fibrotic disease. Numerous evidences have confirmed that miRNA-29 involved in the expression of ECM and regulated organ fibrosis [11]. miRNA-29 as promoters of osteoblast differentiation and apoptosis but suppressors of chondrogenic and osteoclast differentiation, fibrosis, and T cell differentiation [12]. Protective role of estrogen-induced miRNA-29 expression in carbon tetrachloride-induced mouse liver injury [13]. In Crohn's disease fibrosis-reduced expression of the miR-29 family enhances collagen expression in intestinal fibroblasts [14], NF-κB mediated downregulation of miRNA-29 and lower expression of miRNA-29 promoted the deposition of collagens in fibrotic liver [15]. It was found that the expression of miRNA-29 was downregulated in the liver tissue of TX mice with WD liver fibrosis. The expression of miRNA-29 was upregulated with GDFMD intervention. The prediction analysis result showed that miRNA-29 interacted with autophagy gene ULK1 and could inhibit cell autophagy.

Autophagy is an important pathway for protein renewal, which is the most important feature in the process of cell differentiation. Relevant studies have confirmed that autophagy plays an important role in cell reprogramming. Autophagy is essential for removing dysfunctional organelles and protein aggregates and maintaining stem cell homeostasis, including self-renewal, cell differentiation, and somatic reprogramming [16, 17]. Continuous autophagy is conducive to the transformation of fibroblasts into myofibroblasts [18]. Protein remodeling is the key process of cell reprogramming [19]. The loss of autophagy could lead to the accumulation of damaged mitochondria and the self-renewal activity of hematopoietic stem cells [20]. Cellular reprogramming requires rearrangement of the proteome, organelles, and metabolism [21]. A variety of proteins are synthesized and assembled correctly for the activation, and differentiation. Autophagy could remove protein signatures that might hamper reprogramming [22]. The autophagy plays critical roles in stem cell quiescence, activation, differentiation, and self-renewal. Autophagy is a key regulator of stem cell function and how defective stem cell autophagy contributes to degenerative disease, aging, and the generation of cancer stem cells [23]. Autophagy is very critical for multiple cellular processes. Autophagy plays a critical role in bone cell differentiation and function [24]. AFF4 regulates cellular adipogenic differentiation via targeting autophagy [25]. APPL1/Myoferlin downregulation inhibiting autophagy flux, impairing the balance of hMSCs adipogenic and osteogenic differentiation in osteoporosis [26]. Moreover, we will discuss the merits of targeting autophagy as a regenerative medicine strategy to promote stem cell function and improve stem cell-based therapies. Liver myofibroblasts are crucial mediators of ECM deposition in liver fibrosis. They arise mainly from HSCs upon a process termed “activation” [27]. The actived HSCs were cleared from in the injured liver by autophagy inhibitors [9]. HSC activation is followed by an increased autophagic flux. Autophagy inhibition can partially inhibit the transdifferentiation of HSC into myofibroblasts. Autophagy is a possible target in the prevention of HSC activation [28].

Above all, we will further explore the regulatory relationship between GDFMD, autophagy, and liver fibrosis in this work.

## 2. Materials and Methods

### 2.1. The Analysis of Clinical Data

The GDFMD ingredients: Polygonum multiflorum 4 g, Wolfberry 20 g, Sanqi 3 g, Tuckahoe 12 g, Paeony 15 g, Bupleurum 12 g, and Yujin 12 g. These Chinese medicines are provided by the First Affiliated Hospital of Anhui University of Chinese Medicine. Patients should take one dose a day—400 mL decoction in water, which was drinked 200 mL in the morning and evening, respectively.

The clinical indicators of WD patients were collected before and after treatment with GDFMD. The clinical indicators of WD patients were collected before and after treatment with GDFMD. Then the liver injury related indicators ALT, AST, DBIL, IBIL, and TBIL were analyzed. While liver fibrosis indicators Collagen IV and HA were analyzed, the WD patients' 24-h urine ketone was analyzed.

### 2.2. Isolation, Culture, and Treatment of Primary Cells

The wild-type male C57BL/6 J mice (10–12 weeks weighing 20–30 g) were purchased from the Experimental Animal Center of Guangdong Province (Guangzhou, China). Primary HSCs were isolated from the mice liver, and cultured in Dulbecco's modified Eagle's medium (DMEM) with 10% fetal bovine serum (04-001-1ACS, BI). All cells cultured under a humidified 5% (*v*/*v*) CO_2_ atmosphere at 37°C. TGF-β1 is an inducer of HSC activation and liver fibrosis [29]. The HSCs were stimulated with 5 ng/mL TGF-β1 for 48 h. miRNA-29b-3p mimics or inhibitors were transfected into HSCs by lipo2000. All animal experiments were approved by the Animal Care Committee for Animal Research of The First Affiliated Hospital of Anhui University of Chinese Medicine.

### 2.3. CCK8 Assay

The HSCs were seeded into 96 well plates. The HSCs were treated with different conditions for 24 h, when HSCs grew well. Then HSCs were incubated with 10% CCK8 culture medium for 1–2 h. The optical absorption value at 450 nm was detected by microplate reader.

### 2.4. Quantitative Real-Time PCR Assay

Total RNA was extracted from HSCs with TRIzol reagent (Life technogies, 15596018) following the manufacturer's instructions. The concentration of RNA was determined by NanoDrop ND-1000 Spectrophotometer (NanoDrop Tech, USA). Next, the RNA was reverse-transcribed PCR reaction with PrimeScript RT reagent Kit (TaKaRa, RR047A) and PCR products were subjected to electrophoretic analysis. The Novostart SYBR qPCR SuperMix Plus (novoprotein, E096-01B) was used for the real-time PCR analysis of the mRNAs of α-SMA, COL1, MMP2, TIMP1, ULK1, Beclin1, and the β-actin. PCR was performed at 95°C for 1 min, one cycles, and 95°C for 20 s, 60°C for 1 min, 40 cycles. The sense primers and antisense primers were as follow:

5ʹ—GTACCACCATGTACCCAGGC—3ʹ and 5ʹ—GCTGGAAGGTAGACAGCGAA —3ʹ; (mouse α-SMA); 5ʹ—AGCACGTCTGGTTTGGAGAG—3ʹ and 5ʹ—CATTAGGCGCAGGAA GGTCA—3ʹ (mouse COL1); 5ʹ—TAGTACTCTGGAGCGAGGAT—3ʹ and 5ʹ—ACTCCAGT TAAAGGCAGCAT—3ʹ (mouse MMP2); 5ʹ—CACACCAGAGCAGATACCAT—3ʹ and 5ʹ—TTATGACCAGGTCCGAGTTG—3ʹ (mouse TIMP1); 5ʹ—GTACCAGCTCCAGGAAAG TGT—3ʹ and 5ʹ—GTCAATGGCAGTCTGTAGGC—3ʹ (mouse ULK1); 5ʹ—GCTGGAAGA TGTGGAAAAGA—3ʹ and 5ʹ—CTCACTATACTCCCGCTGGTAC—3ʹ (mouse Beclin1); 5ʹ—AGTGTGACGTTGACATCCGT—3ʹ and 5ʹ—TGCTAGGAGCCAGAGCAGTA—3ʹ (mouse β-actin). Relative expression was calculated using the comparative threshold cycle method and determined by the value of 2^–ΔΔCt^.

### 2.5. Western Blot Assay

The protein was extracted from HSCs cells with RIPA lysis buffer (Beyotime Institute of Biotechnology, P0013B), separated by electrophoresis in sodium dodecyl sulfate-polyacrylamide gel. and then transferred to polyvinylidene fluoride membranes (IPVH00010; Millipore). After blocking in 5% nonfat milk for 1 h at RT, the membranes were probed with primary antibody against α-SMA (bs-0189R, bioss), Col1 (bs-10423R, bioss), MMP2 (bsm-51472M, bioss), TIMP1 (bsm-10895M, bioss), ULK1 (4773S, CST), Beclin1 (ab210498, abcam), or GAPDH (TA-08, Zshio), followed by HRP-conjugated Goat anti-rabbit/mouse secondary antibody (ZB-2301/ZB-2305, Zsbio). The blots were visualized with an ECL Kit (34095, Thermo) and densities of target proteins were analyzed by image J software.

### 2.6. Immunofluorescence

The HSCs fixed with 4% paraformaldehyde on glass and the mouse liver tissues Sections were incubated with the anti-LC3B antibody (83506S, 1:200, CST), or anti-*α*-SMA antibody (SC-53142, 1:200, Santa) at 37°C for 2 h, then consecutively incubated with the FITC conjugated goat anti-mouse lgG (B028, 1:200, Ebiogo). DAPI dye was applied for nuclei counterstain. Images were taken with fluorescent microscope (Olympus).

### 2.7. Transmission Electron Microscope (TEM) Assay

The 1 × 10^7^ HSCs were collected by centrifugation, and fixed with 2.5% glutaraldehyde and 1% osmium tetroxide, rinsed in 100 mM sodium phosphate buffer (pH 7.2), dehydrated in ethanol and embedded with Eponate 12 Resin (18005, TED PELLA INC). Ultrathin sections of HSCs were collected on formvar-coated grids and stained with 10% uranyl acetate and 1% lead citrate, and then examined with JEM1400 Transmission Electron Microscope (JEOL, Japan) operated at 80 KV.

### 2.8. The Animal Experiment

ATP7B gene mutation mice, C3He-Atp7btx-J/J TX (10–12 weeks weighing 20–30 g), were purchased from Jackson Laboratory. The GDFMD group mouse were treated with GDFMD by gavage. 1 month later, the liver tissues were obtained, and LC3, α-SMA, and Col1 were detected by immunofluorescence.

### 2.9. Statistical Analysis

All results are represented as the means ± SD of at least three independent experiments. The statistical significance was analyzed by a student's *t*-test for comparison between two groups. *⁣*^*∗*^*p* < 0.05, *⁣*^*∗∗*^*p* < 0.01, and *⁣*^*∗∗∗*^*p* < 0.001 were considered statistically significant. All statistical results were analyzed using GraphPad Priam 8.0.

## 3. Results

### 3.1. Treatment of WD With GDFMD

**Figure 1:**
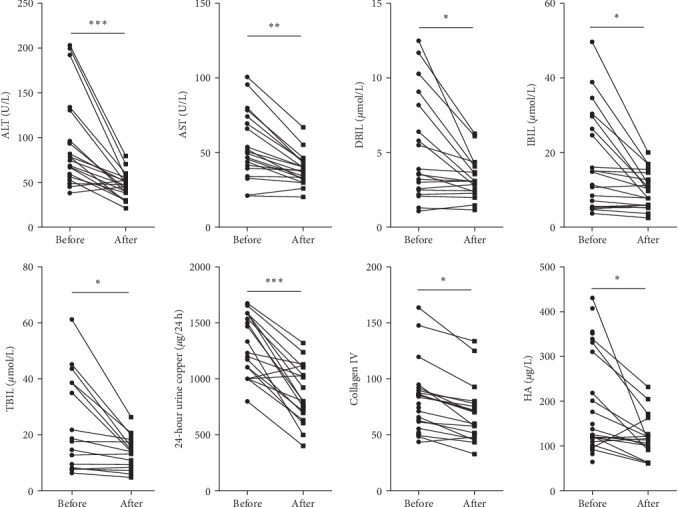
The clinical indicators were detected in WD patients with GDFMD treatment. *⁣*^*∗*^*p* < 0.05; *⁣*^*∗∗*^*p* < 0.01; *⁣*^*∗∗∗*^*p* < 0.001.

This project collected clinical data from 30 WD patients treated with GDFMD. The results showed that liver injury related indicators ALT, AST, DBIL, IBIL, and TBIL were significantly downregulated after GDFMD treatment, while liver fibrosis indicators Collagen IV and HA were significantly downregulated after treatment. The WD patients' 24-h urine ketone significantly decreased with GDFMD treatment (Figure 1).

### 3.2. GDFMD Can Inhibit the Transformation of HSCs Into Myofibroblasts

**Figure 2:**
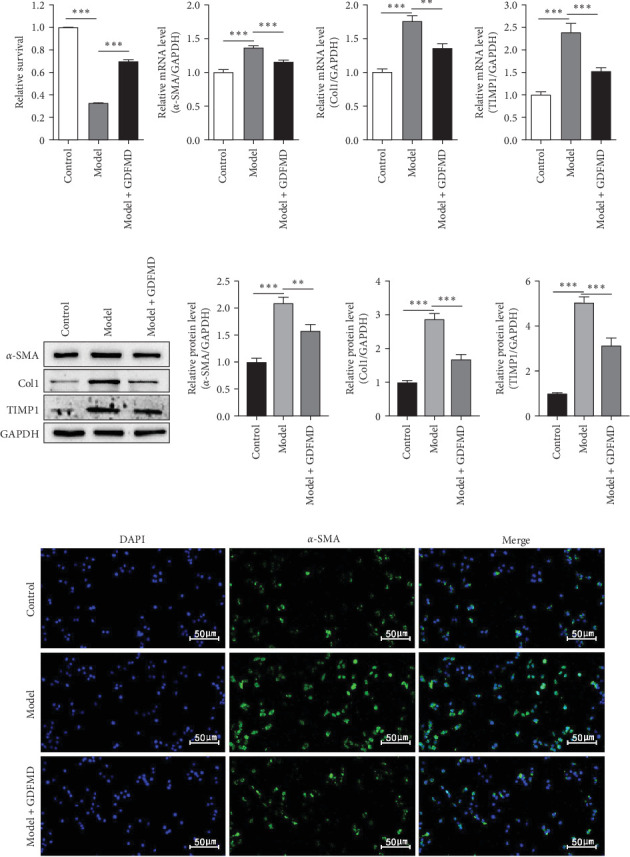
The GDFMD inhibits the transdifferentiation of HSCs into myofibroblasts. (A) With GDFMD treatment, the cell activity were detected by CCK8 assay; (B) The mRNA levels of α-SMA, Col1, and TIMP1were checked by qPCR; and (C) The protein levels of α-SMA, Col1, and TIMP1 were checked by WB; (D) The transdifferentiation ability of HSCs into myofibroblasts was evaluated with anti-α-SMA antibody by IF. *⁣*^*∗∗*^*p* < 0.01; *⁣*^*∗∗∗*^*p* < 0.001.

The HSCs were cultured with TGF-β1 and GDFMD. The cell activity decreased in model group, while increased with GDFMD (Figure 2A). The changes of fibrosis indexes were detected by qPCR and WB. The results showed that after TGF-β1 treatment, the mRNA and protein expression levels of α-SMA, Col1, and TIMP1 increased, while the level of MMP2 decreased (Figure 2). These results indicate that TGF-β1 promoted HSCs transformed into myofibroblasts. Compared with model group, the Model+ GDFMD group results showed that the protein expression levels of α-SMA, Col1, and TIMP1 increased, while the mRNA and protein expression level of MMP2 decreased (Figure 2). The levels of α-SMA is higher in model group, and decreased with GDFMD (Figure 2D). It is indicated that GDFMD could inhibit the transformation of HSCs into myofibroblasts, for that, GDFMD could inhibit the process of liver fibrosis.

### 3.3. GDFMD Promotes Upregulation of miRNA-29b-3p

**Figure 3:**
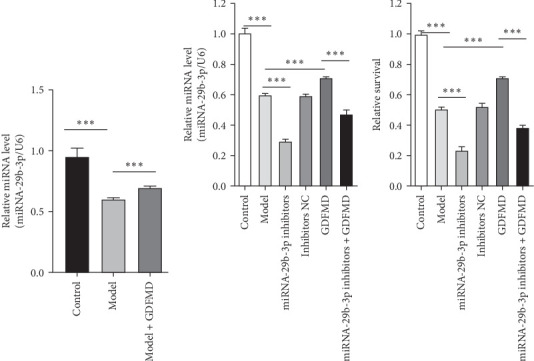
The GDFMD promotes miRNA-29b-3p expression. (A) The miRNA-29b-3p expression levels regulated by GDFMD; (B) With GDFMD or miRNA-29b-3p inhibitors treatment, the miRNA-29b-3p expression levels were detected by qPCR; and (C) With GDFMD or miRNA-29b-3p inhibitors treatment, the HSCs cell activity were detected by CCK8 assay. *⁣*^*∗∗*^*p* < 0.01; *⁣*^*∗∗∗*^*p* < 0.001.

Related studies have shown that miRNA-29b-3p plays an important regulatory role in the progression of fibrosis. The expression of miRNA-29b-3p in HSCs transformed myofibroblasts was down treated with TGF-β1 by qPCR (Figure 3A). The expression of miRNA-29b-3p in HSCs was significantly increased with GDFMD treatment (Figure 3A). The level of miRNA-29b-3p was further promoted by GDFMD, then the level of miRNA-29b-3p downregulate with miRNA-29b-3p inhibitors (Figure 3B). The cell activities rise with GDFMD treatment, and then further inhibited the miRNA-29b-3p expression could reduce cell activity (Figure 3C).

### 3.4. GDFMD Inhibits HSCs Transformed Into Myofibroblasts by Upregulating miRNA-29b-3p

**Figure 4:**
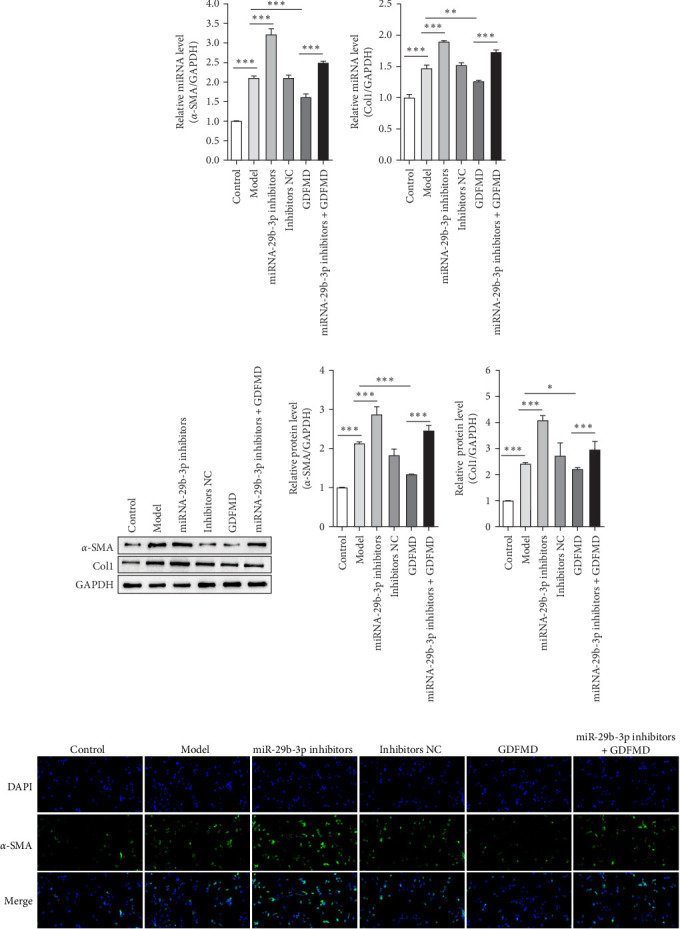
The GDFMD inhibits the transformation of HSCs into myofibroblasts by autophagy with miRNA-29b-3p. (A) The α-SMA and Col1 genes were checked by qPCR. (B) The α-SMA and Col-1 proteins were detected by WB; and (C) The transdifferentiation ability of HSCs into myofibroblasts was evaluated with anti-α-SMA antibody by IF. *⁣*^*∗*^*p* < 0.05; *⁣*^*∗∗*^*p* < 0.01; *⁣*^*∗∗∗*^*p* < 0.001.

The results showed that the downregulation of miRNA-29b-3p expression could reverse the inhibitory effects of GDFMD on fibrosis. Compared with GDFMD group, the α-SMA and COI1 protein levels in GDFMD+miRNA-29b-3p inhibitors group were significantly higher than those in GDFMD group (Figure 4A,B). The myofibroblasts marker α-SMA was checked by IF. The results showed that GDFMT could inhibit the α-SMA protein level, and then miRNA-29b-3p inhibitors further promoted the α-SMA protein level (Figure 4C).

### 3.5. GDFMD Inhibits HSCs Transformed Into Myofibroblasts by Regulating Autophagy

**Figure 5:**
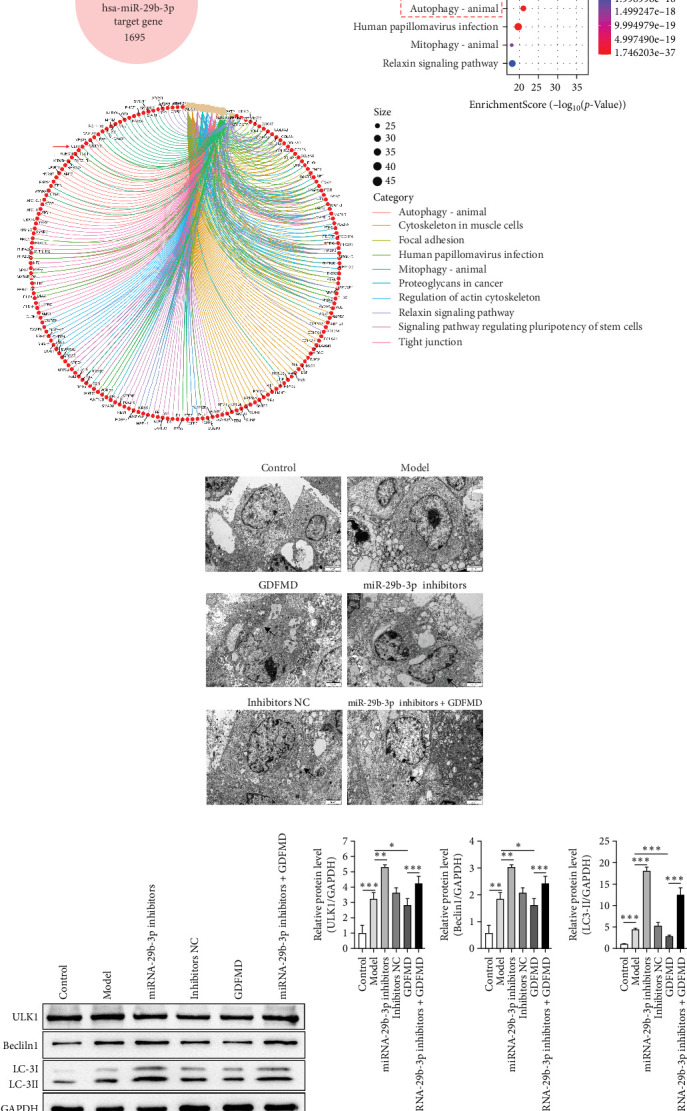
The GDFMD regulates autophagy with miRNA-29b-3p. (A) The relationship of miRNA-29b-3p on cell fibrosis and autophagy pathway was analyzed; (B) The autophagosomes were detected by TEM; and (C) The autophagy proteins were detected by WB. *⁣*^*∗*^*p* < 0.05; *⁣*^*∗∗*^*p* < 0.01; *⁣*^*∗∗∗*^*p* < 0.001.

**Figure 6:**
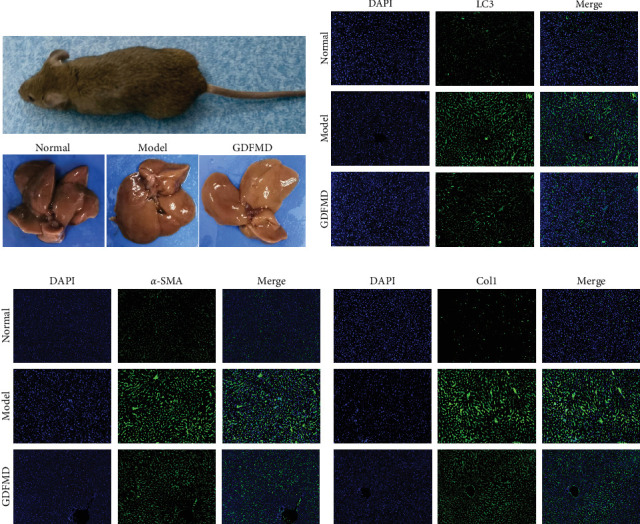
The effects of GDFMD on autophagy and fibrosis in liver tissue. (A) The liver tissues were obtained in different groups; (B) The LC3 protein level was detected by IF; and (C) The α-SMA and Col-1 proteins levels were detected by IF.

The ENCORI database showed that miRNA-29b-3p could target 1886 genes, including 175 fibrosis-related genes, 32 autophagy related genes, and 16 genes involved in both autophagy and fibrosis. The autophagy gene ULK1 was an important target gene of miRNA-29b-3p (Figure 5A). Studies have confirmed that autophagy can promote the transformation of HSCs into myofibroblasts. It has been previously confirmed that GDFMD can inhibit the transformation of HSCs into myofibroblasts. To further confirm that GDFMD plays a regulatory role by inhibiting autophagy through miRNA-29b-3p. TEM results further confirmed that autophagy increased in GDFMD+miRNA-29b-3p inhibitors group (Figure 5B). In this part, the level of miRNA-29b-3p was further inhibited in GDFMD treated cells. The WB results showed that compared with GDFMD group, the expression of ULK1, Beclin1, and LC-3 increased in GDFMD+miRNA-29b-3p inhibitors group (Figure 5C). Compared with GDFMD group, The LC-3 protein level is increase in GDFMD+miRNA-29b-3p inhibitors group (Figure 5C). It is demonstrated that GDFMD inhibits autophagy of HSCs with miRNA-29b-3p. The IHC result showed that GDFMD inhibited the protein levels of LC3, α-SMA, and COL1 in vivo (Figure 6A–C).

## 4. Discussion

Liver fibrosis is the main pathological change of the liver in almost every WD patient, and it is the necessary stage to progress to cirrhosis [30]. Related studies have confirmed that HSCs play an important role in the repair of liver injury [31]. HSCs are a kind of non-parenchymal cells unique to the liver, accounting for about 33% of the total number of non-parenchymal cells, and 1.4% of the total volume of the liver. HSCs with a mesenchymal origin involved in ECM homeostasis. The HSCs activate and acquire myofibroblastic features, secrete ECM participating under inflammatory conditions [6]. Myofibroblasts is an important driver of liver fibrosis. In response to injury, the excessive or sustained activation of myofibroblasts promotes its proliferation, remodeling, and maturation, secretes a large amount of ECM, causes tissue and organ fibrosis, and leads to organ failure [32]. Therefore, regulating tissue repair by controlling the role of myofibroblasts has become the current focus of attention.

Our clinical and animal studies have confirmed that GDFMD can effectively improve the level of liver fibrosis in patients with WD. However, its related mechanism needs further study. The vitro liver fibrosis model was established with HSCs induced by TGF-β1. The results showed that the α-SMA expression was upregulated in the model group. It indicates that the model is successfully constructed. Further, the model was treated with GDFMD. The expression of α-SMA was downregulated, and the levels of tissue inhibitors of metalloproteinase-1 (TIMP1) and COL1 secreted by myofibroblasts were decreased. α-SMA is currently considered as a marker of myofibroblasts [33]. When liver injury continues, HSCs derived myofibroblasts continuously produce collagen (COL1) and TIMP1. Increased fibrogenesis and decreased fibrinolysis are two important factors leading to excessive accumulation of ECM [34]. These results indicate that GDFMD could inhibit the transformation of HSCs into myofibroblasts.

Previous studies have found that the expression of miRNA-29b-3p was downregulated in WD mice and significantly upregulated after GDFMD treatment. GDFMD could upregulate the expression of miRNA-29b-3p.

Related studies have confirmed that autophagy plays an important role in the process of cell differentiation [35]. In the process of cell differentiation, autophagy control mesenchymal stem cell (MSC) differentiation by proteomic reprogramming [36]. Autophagy is an important intervention way in the process that the HSCs transdifferentiate into myofibroblasts. For this reason, we guess that whether miRNA-29b-3p plays a regulatory role by interfering with autophagy. We predicted that miRNA-29b-3p interacts with ULK1. With miRNA-29b-3p inhibitors treatment, the expressions of ULK1, Beclin1, and LC3 proteins were upregulated. These results indicate that miRNA-29b-3p can effectively inhibit ULK1 dependent autophagy.

**Figure 7:**
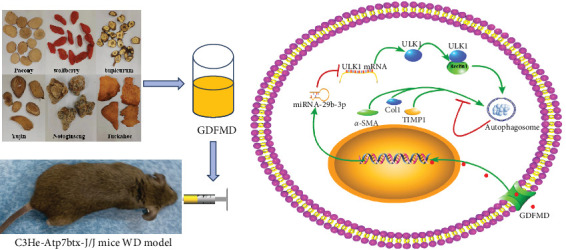
The mechanism of GDFMD in WD. (A) The GDFMD acquisition process and gavage schematic diagram; (B) The mechanism diagram of GDFMD in the treatment of WD disease.

To further prove that GDFMD regulates autophagy by miRNA-29b-3p. We further performed miRNA-29b-3p interference on GDFMD group. The results showed that after GDFMD treatment, the expression of miRNA-29b-3p was upregulated, the expression of autophagy related proteins were downregulated, and autophagosomes were reduced. Then treatment with miRNA-29b-3p inhibitors, the expression of miRNA-29b-3p was downregulated, the expression of autophagy related proteins could upregulate, and autophagosomes were increased. The protein level of LC3, α-SMA, and COL1 are decreased with GDFMD in vivo. So that, the GDFMD inhibited the tissue fibrosis by autophagy in vivo (Figure 7A). In recent years, miRNA has attracted much attention and been widely studied in many diseases. Numerous evidences have confirmed that miRNA-29 involved in the organ fibrosis and cell differentiation [11–15]. However, RNA faces challenges in transformation applications due to its poor stability. It is believed that with the progress of technology, this problem can be solved.

## 5. Conclusion

GDFMD inhibits autophagy by upregulating miRNA-29b-3 and inhibits the transdifferentiation of HSCs into myofibroblasts to achieve the treating liver fibrosis of WD patients (Figure 7B).

## Data Availability

The datasets generated during and/or analysed during the current study are available from the corresponding author on reasonable request.
